# Single-Cell Approaches to Profile the Response to Immune Checkpoint Inhibitors

**DOI:** 10.3389/fimmu.2020.00490

**Published:** 2020-03-20

**Authors:** Lara Gibellini, Sara De Biasi, Camillo Porta, Domenico Lo Tartaro, Roberta Depenni, Giovanni Pellacani, Roberto Sabbatini, Andrea Cossarizza

**Affiliations:** ^1^Department of Medical and Surgical Sciences for Children and Adults, University of Modena and Reggio Emilia, Modena, Italy; ^2^Department of Internal Medicine and Therapeutics, Division of Translational Oncology, IRCCS Istituti Clinici Scientifici Maugeri, University of Pavia, Pavia, Italy; ^3^Department of Oncology, Hematology, University of Modena and Reggio Emilia, Modena, Italy; ^4^Department of Surgery, Medicine, Dentistry and Morphological Sciences, University of Modena and Reggio Emilia, Modena, Italy; ^5^Section of Modena, Istituto Nazionale per le Ricerche Cardiovascolari, Bologna, Italy

**Keywords:** immunotherapy, immune checkpoint, single-cell technologies, cancer, immune system

## Abstract

Novel treatments based upon the use of immune checkpoint inhibitors have an impressive efficacy in different types of cancer. Unfortunately, most patients do not derive benefit or lasting responses, and the reasons for the lack of therapeutic success are not known. Over the past two decades, a pressing need to deeply profile either the tumor microenvironment or cells responsible for the immune response has led investigators to integrate data obtained from traditional approaches with those obtained with new, more sophisticated, single-cell technologies, including high parameter flow cytometry, single-cell sequencing and high resolution imaging. The introduction and use of these technologies had, and still have a prominent impact in the field of cancer immunotherapy, allowing delving deeper into the molecular and cellular crosstalk between cancer and immune system, and fostering the identification of predictive biomarkers of response. In this review, besides the molecular and cellular cancer-immune system interactions, we are discussing how cutting-edge single-cell approaches are helping to point out the heterogeneity of immune cells in the tumor microenvironment and in blood.

## Introduction

Immune checkpoints are critical regulators of the immune system which modulate the duration and amplitude of immune responses to maintain self-tolerance and prevent autoimmunity. Among immune checkpoints, cytotoxic T lymphocyte antigen-4 (CTLA-4, or CD152), programmed death-1 (PD-1, or CD279) and programmed death ligand-1 (PD-L1, or CD274) have been intensively studied and antibodies against these molecules have been developed to successfully reinvigorate T cell functions and provide a durable immune response. Antibodies against immune checkpoints have demonstrated impressive efficacy, and now constitute the backbone of systemic therapy in different malignancies (1).

Despite considerable advancements in clinical care, epidemiologic data and ongoing clinical trials suggest that most patients receiving immune checkpoint inhibitors (ICI) do not derive benefit or stable and lasting responses. The mechanisms at the basis of this lack of responsiveness are multiple, and still not completely known. Over the past years, accumulating evidence suggested that the elevated neoantigen load (i.e., the number of antigens actually targeted by T cells) may have a robust relationship with the response to ICI (2). In particular, the more is the neoantigen load, the better is the response to therapy. However, the intensity and efficacy of the immune response can vary upon neoantigens' clonality. It seems that neoantigens derived from clonal mutations, which appear early during tumorigenesis, may elicit more effective tumor responses than neoantigens derived from subclonal mutations, which are acquired later in tumorigenesis (3). This means that intratumoral heterogeneity may impact the response to ICI. Moreover, several biophysical matters occur in the binding and recognition between peptide-MHC complex and T cell receptor (TCR) (4), and current prediction algorithms are still unable to precisely define TCR binding capacity for specific neoantigens (2). An additional layer of complexity originates from the fact that T cells, which are the main mediator of anti-tumor immunity, are extremely heterogenous in the tumor microenvironment (TME), and that beyond T cells many other types of immune cells are present in the tumor tissue that could affect response to ICI (4). Furthermore, an anticancer immune response may be impaired also by a number of other factors, mainly immune cells polarized toward an immune suppressive phenotype (5).

Taken together, these observations suggest that most, if not all, of these components are involved in the clinical response to ICI, and that the identification of the mechanism(s) at the basis of such response is crucial, both to provide important insights into the molecular and cellular crosstalk between cancer and immune system, and possibly foster the identification of predictive biomarkers of response (6). In this scenario, recently several novel single-cell technologies have been used to draw an in-depth characterization of tumor and immune system ecosystems in different malignancies. Here, we first describe the interactions between tumor and immune cells and then give an overview of the cutting-edge single-cell approaches mainly used to interrogate cancer immunity both in the tumor microenvironment and in the blood. We also cover and discuss how single-cell analysis have revealed the vast heterogeneity characterizing intra-tumoral immune cells, mainly T cells, and how this knowledge is critical to understand the role of different cell states and phenotypes in the response to immune checkpoint inhibitors.

## Immune System and Cancer

### Cancer Immunosurveillance and Immunoediting

The long-standing theory of immune surveillance suggests that cells and tissues are regularly monitored by the immune system, which is responsible for recognizing and eliminating the vast majority of nascent cancer cells. The interactions between cancer and the immune system are regulated by a complex network of biological pathways, and start during the early steps of carcinogenesis, when normal cells acquire biological capabilities which allow them to evolve progressively to a neoplastic state. Such capabilities are commonly known as hallmarks of cancer, and among them the ability to evade immune system is crucial to guarantee cancer cell survival and tumor progression (7).

Over the past years, accumulating evidences, both from murine models and clinical epidemiology, have validated the concept of cancer immunosurveillance, demonstrating that the immune response acts as a barrier to tumor development and progression, and is a critical determinant of susceptibility to tumors (8–13). This action is exerted through at least three distinct mechanisms: (i) protection of the host from viral infections, and thus suppression of tumors of viral etiology; (ii) prevention or resolution of inflammation, which facilitates tumorigenesis; (iii) identification and elimination of cancer cells, in certain tissues and on the basis of the expression of tumor-specific antigens (14). In particular, deficiencies in the number or functionality of CD8+ cytotoxic T lymphocytes (CTLs), CD4+ type-1 T helper (Th1) cells, natural killer (NK) cells, natural killer T (NKT) cells, B cells, or γδ T cells lead to increased susceptibility to carcinogen-induced tumors and spontaneous tumors development (9, 11, 15, 16). Similarly, unsensitivity to interferons (IFNs) or lack of perforin, interleukin (IL)-12, tumor necrosis factor (TNF)-α or IL-1β are associated with increased tumor susceptibility (10, 11, 17–20).

Reinforcing this notion, clinical epidemiology supports the existence of antitumoral immune response in some types of cancer. Firstly, evidence for immunosurveillance can be found in patients with acquired immunodeficiencies, like that caused by the human immunodeficiency virus (HIV), the cause of AIDS, who have an increased frequency of virus-associated malignancies, including Kaposi's sarcoma, lymphomas, urogenital cancers, and cervical cancers due to different strains of papillomavirus (21). Secondly, higher cancer prevalence has been observed in transplanted recipients treated with immunosuppressive drugs. Immunosuppression to prevent transplant rejection is associated with a 3- to 100-fold increased risk of developing certain types of cancer, mainly lymphomas (22). However, solid tumors with no viral etiology also occur with increased frequency. For example, patients receiving renal transplant have a 3-fold increase in the incidence of cancers respect to the general population, and a 200-fold increase of skin cancers (23). Patients with liver transplant also display a greater incidence of malignancies, including head and neck cancers, and skin cancers (24, 25).

Along with clinical epidemiology, other evidences support the theory of cancer immunosurveillance, including the identification of tumor antigens and of antibodies against those antigens. In cancer patients, humoral immune response has been detected against more than a hundred tumor-associated antigens, thus indicating that the immune system is well able to fight against cancer (26). However, whether or not the identification and quantification of these antibodies has a clear diagnostic and/or prognostic relevance is still unclear. Other spontaneous immune responses against cancer cells have been described in paraneoplastic autoimmune syndromes, caused by the activation of an immune response against self-antigens expressed on cancer cells. Paraneoplastic autoimmune syndromes are often caused by cross-reactivity between the anti-tumor immune response and antigens present in the nervous system, and the onset of neurologic symptoms typically precedes the diagnosis of a formerly undetected tumor (27).

Advancements made all over the past two decades have demonstrated that the immune system not only defends the host against tumor development, but also edits tumor immunogenicity, in a process referred to as cancer immunoediting. In its most complicated form, cancer immunoediting proceeds through three phases, termed elimination, equilibrium and escape. During the elimination phase, both innate and adaptive immune system collaborate to recognize and kill neoplastic cells. Cancer clones which survive the elimination phase can then progress through the equilibrium phase, in which tumor growth is limited, but cellular immunogenicity is edited by the adaptive immune system, mainly lymphocytes. During this phase, the pressure of the immune system together with the genetic instability of tumor cells can lead to the selection of neoplastic subclones with low immunogenicity, which can enter into the escape phase and evade the immune recognition. A large number of mechanisms operate to enable tumor immune escape by interfering with almost every step required to generate an effective immune response, i.e., the: (i) tumor capacity to downregulate antigens and/or MHC I; (ii) tumor expression and/or secretion of immunosuppressive molecules and/or antiphagocytosis signals; (iii) tumor modulation of lymphocytes' metabolism; (iv) recruitment of immune cells that actively mediate tolerance, or even promote tumor growth (28).

Since Virchow noted the presence of lymphocyte infiltration in solid tumors in 1863 (29), additional support for cancer immunosurveillance and immunoediting is evident in uncountable reports that describe the presence of immune cells infiltrating the tumors, and that correlate their frequency with patient prognosis. The presence of T cells inside tumors was observed, in late 1990's, in patients with melanoma, and then described for several other malignancies, including ovarian, colorectal (CRC) or lung cancer (30–36). From then on, a great attention has been paid to investigate the role of tumor-infiltrating lymphocytes (TILs). This effort resulted in the identification of TILs frequency as a *bona fide* indicator of improved prognosis and increased overall survival for several types of tumors.

Recent advancements in the characterization of the immune context within the tumor microenvironment have revealed that different classes of the so-called tumor immune environment (TIME) exist that are associated to tumor initiation and could affect the response to therapies (37). The TIME varies greatly across individuals and over distinct cancers. However, despite variability, two main classes can be described, which differ on the basis of composition, functional status and spatial distribution of immune cells. Infiltrated-excluded TIMEs are populated by immune cells mainly along the tumor margins, and are relatively poor of CTLs in the tumor core (37). Moreover, CTLs from this kind of TIME typically display low expression of activation or cytotoxicity markers, including granzyme(GZM)-B and IFN-γ (37). Conversely, infiltrated-inflamed TIMEs are characterized by large immune infiltration among neoplastic cells, with a high frequency of CTLs expressing GZM-B, IFN-γ, and PD-1. In some cases, infiltrated-inflamed TIMEs contain compartments which resemble tertiary lymphoid structures (TLSs), and act as sites of lymphoid recruitment and immune activation (38). Such compartments are generally located at the invasive tumor margin and in the stroma, and include naïve and activated T cells, regulatory T (Treg) cells, B cells and dendritic cells (DCs) (37). Over the past years, the immune network of the TME has become a focus of cancer research and therapeutics development, and the need to understand its great complexity and diversity in this context is now compelling.

### Immune Checkpoints and Their Inhibitors

Immune checkpoints are molecules expressed on T cell plasma membrane able to inhibit or activate the development or execution of effector functions exerted by cytotoxic or pro-inflammatory T cells. Among immune checkpoints, CTLA-4 and PD-1 have been most actively studied in the field of clinical cancer immunotherapy.

CTLA-4 and CD28 are homologous molecules expressed by CD4+ and CD8+ T cells, which mediate antagonistic functions in T cell activation, and share two ligands, namely B7-1 (CD80) and B7-2 (CD86), expressed on antigen-presenting cells (APCs). CD28 interacts with the CD80 dimer with relatively high affinity and the CD86 monomer with lower affinity, to mediate T cell activation in conjunction with TCR signals. Conversely, CTLA-4 interacts with both ligands with higher affinity and avidity than CD28, to inhibit T cell activation. CTLA-4 is constitutively expressed on Treg cells or induced following T-cell activation via CD28 and TCR signaling (39). The humanized anti-CTLA-4 antibody ipilimumab was approved by the United States Food and Drug Administration (FDA) in 2011. It blocks the interaction between CTLA-4 and its ligands expressed by APCs, thereby preventing the transmission of inhibitory signals to CTLA-4-expressing T cells. Although the blocking of inhibitory signals is the main mechanistic contributor to ipilimumab functions, other still poorly known mechanisms are involved. For example, the effects of anti-CTLA-4 on Treg is still matter of debate. Indeed, the binding of CTLA-4 by ipilimumab on Treg within the tumor tissue would likely promote Treg depletion by antibody-dependent cellular cytotoxicity (ADCC) and phagocytosis by NK cells and macrophages (40, 41). Recently it was found that both ipilimumab and tremelimumab, another anti-CTLA-4 drug, increase infiltration of intratumoral CD4+ and CD8+ T cells without significantly changing or depleting FOXP3+ cells within the TME (42). Nonetheless, regardless the mechanism of action, ipilimumab demonstrated impressive anti-tumor activity in several clinical settings in metastatic melanoma (43, 44).

Along with CTLA-4, the PD-1/PD-L1 system constitutes another immune checkpoint pathway mainly operating by controlling immune homeostasis. However, while transient expression of PD-1 is a feature of normal T lymphocyte activation, persistent antigen exposure leads to a sustained expression of PD-1 with a gradual loss of effector functions which are characteristic of exhausted T cell (45). PD-1 mediates an inhibitory signal in T cells after binding to its ligands, PD-L1 and PD-L2, which are expressed on APCs and cancer cells (46). The blockade of PD-1/PD-L1 pathway with anti-PD-1 or anti-PD-L1 antibodies, can successfully reinvigorate T cell functions and provide a durable response in different malignancies. There are currently six inhibitors of the PD-1/PD-L1 pathway, namely nivolumab, pembrolizumab, cemiplimab (directed against PD-1), and atezolizumab, avelumab and durvalumab (directed against PD-L1), which have been approved by the FDA for the treatment of tumors like melanoma, lung cancer, renal-cell carcinoma (RCC), microsatellite instability-high CRC, classical Hodgkin lymphoma, head and neck squamous cell cancer (HNSCC), hepatocellular carcinoma (HCC), bladder cancer, gastro-oesophageal cancer, and unresectable or metastatic, microsatellite instability-high or mismatch repair deficient solid tumors (47).

The best examples of stable response are those observed in patients with advanced melanoma. In these patients, it was reported that 3-year overall survival was 34 and 52% for ipilimumab and nivolumab, respectively (48). The 3-year overall survival was 60, 55, 41% for nivolumab plus ipilimumab, nivolumab alone, or ipilimumab alone (49). In advanced RCC, the 2-year overall survival of patients treated with nivolumab plus ipilimumab was 28%, and complete responses were 11% (50–52). In other cancers, responses to immune checkpoint monotherapies were not as impressive as in melanoma. This means that despite considerable advancements in clinical care of some tumors, epidemiologic data and ongoing clinical trials suggest that most of the patients receiving ICI do not derive benefit or durable responses, and the mechanisms at the basis of this lack of responsiveness are multiple and still not completely known.

## Single-Cell Approaches to Immune Profile

Over the past two decades, a pressing need to deeply profile the TIME has led investigators to complement data obtained from traditional approaches, like immunohistochemistry, basic flow cytometry or measurements on bulk populations of cells, with data obtained with novel, more sophisticated, single-cell technologies. To date, a vast array of single-cell approaches, including high-parameter flow cytometry, deep sequencing, and high-resolution imaging are available to unmask cellular heterogeneity and to try to identify actionable hallmarks of efficient anticancer immunotherapy. In Table 1 a general overview of single-cell technologies is provided together with their advantages and disadvantages.

**Table 1:** Advantages and disadvantages of the cutting-edge single-cell technologies to profile cancer immunity.

**Methodology**	**Advantages**	**Disadvantages**	**References**
Flow cytometry	• Evaluation of protein, RNA and DNA at a single-cell level simultaneously; • Easy and fast sample preparation; • Acquisition of sample is high-throughput and fast; • A plethora of unsupervised and supervised data analysis methods available (global data structure, cellular progression, cellular diversity, signaling network inference, correlative/predictive features of clinical outcome or sample type); • Possible to sort cells and perform further studies; • The cost is cheap (more or less, US $ 0.10 per cell).	• Limit to 30-parameters at time; • Spillover among different fluorescences; • Quality control of the data needed; • High level of expertise is needed to analyze data; • No information on tissue structure; • Acquisition of samples must occur in a few hours after staining due to photo bleaching.	(53)
Mass cytometry	• Evaluation of protein, RNA and DNA at a single cell level simultaneously (up to 40 parameters—theoretically around 100); • Sample preparation is fast; • Acquisition of sample is high-throughput; • Metal-tagged samples can be run up to 2 weeks after staining without notable loss of signal and can be cryopreserved up to 1 month without affecting the data quality or staining integrity of both surface and intracellular markers; • A plethora of unsupervised and supervised data analysis methods available (global data structure, cellular progression, cellular diversity, signaling network inference, correlative/predictive features of clinical outcome or sample type).	• Sample acquisition is not fast; • Difficult to measure molecules that are expressed at very low levels; • Quality control of the data needed; • Spillover between close isotopes; • High level of expertise needed to analyse data; • No information on tissue structure; • Impossible to recover living cells after analysis; • The cost is much higher than fluorescence-based flow cytometry (more or less, several US dollars per cell).	(54)
Image-flow cytometry	• Evaluation of protein, RNA and DNA at a single cell level simultaneously (up to 12 parameters); • Easy and fast sample preparation; • Up to 10 fluorescent images per cell; • Images up to 60x magnification; • Detailed localization of signal from fluorescent probes.	• Sample acquisition is not fast; • No information on tissue structure; • High expertise is needed to analyse data; • Only few software used to analyse data; • Not possible to perform unsupervised analysis.	(55)
Histo-cytometry	• Technology is based on multiplexed antibody staining, tiled high-resolution confocal microscopy, voxel gating, volumetric cell rendering, and quantitative analysis; • Gain positional and quantitative information on complex cellular subsets/phenotypes (defined by multiple markers) directly in tissue sections; • Very high-resolution imaging and accurate signal 3D allocation.	• 6–8 colors/parameters; • Spillover between fluorochromes; • Due to the lack of molecular level resolution, imaging does not spatially separate neighboring fluorescent molecules, instead colocalizing them to the same voxel (volumetric pixel); • Software dedicated to imaging; • Low- throughput.	(56)
Imaging mass cytometry	• Analytical platforms that successfully couple high-density analysis by mass cytometry to conventional histology; • Comprehensive exploration of individual cell phenotypes, cell–cell interactions, microenvironments, and morphological structures within intact tissues.	• 1 μm spot size • Sample preparation is slow and needs a lot of technical advices; • The rate of image acquisition by laser ablation is slow (1.5 mm^2^ in 2 h), and sets a practical limit to the extent to which a slide can be scanned; • Many tissue markers of clinical importance show considerable intratumoral heterogeneity in their distribution patterns; • Data analysis remains challenging, and is performed by particular and dedicated software (like HistoCAT).	(57–59)
Single-cell RNA sequencing	• Different methods developed in recent years allow to investigate single-cell transcriptomics; • Two low-throughput plate-based methods (Smart-seq2 and CEL-Seq2) and five high-throughput methods (10x Chromium, Drop-seq, Seq-Well, inDrops, and sci-RNA-seq); • Standardized and optimized protocols; • Very high-throughput; • A plethora of data analysis methods available (global data structure, cellular progression, cellular diversity, signaling network inference, network reconstruction); • On the basis of the type of sequencing it is possible to identify cell clonality, allelic expression, alternative splicing, RNA editing; • 2,000–6,000 genes per cell for primary cells if SMART-seq2 is used; 1,000–3,000 genes if Drop-seq or InDrop is used;	• Long procedures to prepare cDNA libraries; • Sample preparation is long (2 days of protocol); • High cost of single cell sequencing (thousands of US $ per sample); • Data analysis requires the use of highly advanced bioinformatics methods; • Quality control, normalization and imputation needed; • Due to technical limitations and biological factors, scRNA-seq data have some background, and are more complex than bulk RNA-seq data. • The high variability of scRNA-seq data raises computational challenges in data analysis.	(60–62)
	• Low cost of sample preparation: $3–6 per well (if SMART-seq2 protocol is used); $0.05 per cell (if DropSeq or InDrop protocol is applied).		
Single-cell ATAC sequencing	• It interrogates the genome for accessibility to DNA binding proteins in a single experiment; such challenge emphasizes the need for informative features to assess cell heterogeneity at the chromatin level; • scATAC-seq experiments sample DNA, compared to transcriptomic (scRNA-seq) data; • Single-cell ATAC libraries are created from single cells that have been exposed to the Tn5 transposase using one of the following protocols: Single cells are individually barcoded by a split-and-pool approach where unique barcodes added at each step can be used to identify reads originating from each cell, microfluidic droplet-based technologies are used to extract and label DNA from each cell, or each single cell is deposited into a multi-well plate for library preparation. • A plethora of data analysis methods available.	• Sample preparation is long (2 days of protocol); • Data analysis for expert requires the use of bioinformatics methods.	(61, 63)

### High-Parameter Flow Cytometry

Last advances in proteomics and genomics are paving the way to comprehend the complexity and the heterogeneity of billions of specialized immune cells in cancer patients. For decades, immunologists relied mainly on flow cytometry, the first single-cell technique that now allows to study the expression and density of up to 30–40 antigens in a single-cell level. Flow cytometry is very popular technique used to measure physical and chemical characteristics of a population of cells/particles suspended in a fluid, and is routinely used both in basic research, and in clinical practice to perform cell count, determine cell phenotypes and functions allowing the monitoring of immune features in pathophysiological settings (64, 65). Flow cytometry is unmatched for its high throughput as several million cells can be analyzed in a few minutes. In addition, cells can be sorted achieving pure cell populations to perform further functional, metabolic and molecular analyses (66). Sample preparation for flow cytometry is relatively fast, but setting up a flow cytometry panel that includes 28–30 parameters takes a lot of time because of the need to optimize spectral overlap between fluorophores, and to choose best antibodies. These issues can be overcome by following precise rules applicable to panel design, and optimized panels published such as Optimized Multicolor Immunophenotyping Panels (OMIPs) (67–72). Together with fluorescent flow cytometry, mass cytometry (also called CyTOF—Cytometry by Time-Of-Flight) is a technology that allows simultaneous analysis of more than 40 different molecules, including cytokines and transcription factors, with minimal compensation (53, 54). This technique exploits the use of monoclonal antibodies conjugated with heavy-metal isotopes to stain cells and quadrupole time-of-flight mass spectrometer to perform the detection (73). Mass spectrometry is able to discriminate isotopes of different atomic weights with high accuracy, enabling more features to be assayed at the same time, so the quantity of reporter ions in a particular mass channel represents the marker expression with little signal overlap between parameters.

Among high-parameter single-cell technologies, at present flow cytometry is the gold standard. However, it reveals the different percentages of cell populations in different pathophysiological onsets barely identifying different clones (74). Flow cytometry perfectly captures the phenotype of cells, but fails to snap their biological complexity. The spectrum of phenotypic diversity of immune cells within the TME and in blood can better be appreciated by analyses at the single-cell level to explore cellular heterogeneity, in terms of gene expression and chromatin accessibility, that often confounds biomolecular variation from multi-omics approaches in bulk. Recently, the adaptation of high-parameter flow cytometry to imaging platforms took to the development of two promising technologies, known as Imaging Mass Cytometry (IMC), Co-detection by Indexing (CODEX) and multiepitope-ligand-cartography (MELC) (55–57, 75, 76). The former is used to process tissues, employs antibodies tagged with lanthanides and combines a high resolution laser ablation system with CyTOF (77). IMC enables the study of cell-cell interactions and of intercellular networks, thus providing information regarding the spatial distribution of cells within a tissue (57–59). CODEX employs oligonucleotide-conjugated antibodies. Although these technologies have not yet been applied to dissect immune responses in the field of cancer immunotherapy, the power will be used to investigate the role of immune cells in the TME.

### Single-Cell RNA Sequencing

Single-cell RNA sequencing (scRNA-seq) technology provide a transformative view of cell-type-specific gene expression and allows to analyze hundreds of messenger RNAs (mRNAs) in a single experiment, enabling the reconstruction of a high-resolution map of cells according to their molecular signature (66). The first example of single-cell digital gene expression profiling was published in 2009, and since then on, a continuous effort has been made to improve experimental protocols and bioinformatics pipelines, which are essential to process data (60, 78, 79). A canonical scRNA-seq protocol involves several steps, including single cell isolation, cell lysis to obtain RNA, reverse transcription into the first-strand cDNA, second-strand synthesis, cDNA amplification, and sequencing (60–62).

Although single cells can be isolated by different techniques, the use of microdroplet-based microfluidics is now widely diffused among the majority of commercial platforms and allows the isolation of individual cells into aqueous droplets in a continuous oil phase. In every droplet, cells are lysed in a hypotonic buffer, and mRNAs are captured by poly-dT primers. When reverse transcription takes place, cDNA molecules originated from a given individual cells are identified by using short DNA barcode tags. Then, second strands are generated, and the small amount of synthesized double-strand cDNA is amplified by means of conventional polymerase chain reaction (PCR) or *in vitro* transcription, depending on the technology. Some protocols improved read coverage across transcripts, which significantly enhances detailed analyses of alternative transcript isoforms and identification of single nucleotide polymorphisms (SNPs) with high sensitivity and accuracy (80). Eventually, sequencing is performed, and once reads are obtained, data are pre-processed and analyzed through clustering of cells, classification and cell trajectory assignment (78, 79). Concerning T cells, during the last years, several algorithms have also been developed to utilize scRNA-seq data to reconstitute TCR information. TCR is a heterodimer composed of two chains, α and β, which result from genetic recombination of the V(D)J genes, and is responsible for the specificity of each T cell against cognate antigens. The diversity of TCRαβ repertoire is associated with efficient protection against several pathogens (81), and more recently, the clonality of both peripheral blood and tumor TCRαβ repertoire has also been associated with improved clinical outcome under anti-PD-1 or anti-CTLA-4 immunotherapy (82–84).

Despite its numerous pros and great potential, scRNA-seq suffers from the caveat that mRNA and protein expression do not always directly correlate. For this reason, recent technological advances have been made to capture new cell types with a better resolution, and to detect simultaneously mRNAs and proteins. For example, Cellular Indexing of Transcriptomes and Epitopes by Sequencing (CITE-seq), RNA Expression and Protein Sequencing (REAP-seq), Antibody sequencing (Ab-seq) enable the measurement of proteins and mRNAs in individual cells, by using antibodies labeled with DNA barcodes instead of fluorochromes, thus avoiding the limitations dictated by the possible spectral overlap of fluorescent signals (85–87). Quantifying proteins together with mRNAs allows to overcome the lack of correlation that sometimes exists between mRNA and protein levels, thus providing a more readout of cellular phenotype, at the single-cell level. Indeed, proteins, not mRNAs, are the real targets of drugs, and mRNA abundance cannot necessarily resemble protein abundance (85). Moreover, in certain settings, the measurement of protein abundance is more sensitive for markers with low levels of mRNA transcripts (85). Thus, CITE-seq, REAP-seq, and Abseq give an unbiased view of the mRNA and protein profile at the single-cell level, which is necessary to precisely identify cellular function, and provide important insights into the pathophysiology of multiple disorders. However, sample preparation requires more than 2 days and cells need to be fixed or lysed, therefore excluding the possibility to perform further analysis (85–87).

Another possibility to investigate both mRNA and proteins is the combination of scRNA-seq and high-parameter flow cytometry. The combinatorial use of scRNA-seq and high-parameter flow cytometry in the same sample would likely have a huge impact in the field of immunotherapy, as is associated with unique advantages to each method together with the advantage of using both methodologies. Whereas each technology uses unsupervised clustering to identify different populations, scRNA-seq is totally unbiased as it analyses the expression of thousands of genes. Conversely, high-parameter flow-cytometry looks at 30–40 markers that are pre-selected based on *a priori* knowledge. Also, scRNA-seq allows transcriptomic analysis between individual cell subsets, including the use of Gene Set Enrichment Analysis (GSEA) and comparisons to human datasets. However, as already described, mRNA and protein do not always correlate, meaning that the information on protein expression delivered by high-parameter flow-cytometry is also central. However, to date, a few studies reported the combination of scRNA-seq and CyTOF to profile the tumor immune microenvironment (88, 89).

### Single-Cell ATAC-Seq

The Assay for Transposase-Accessible Chromatin using sequencing (ATAC-seq) is a method for assessing genome-wide chromatin accessibility. ATAC-seq identifies accessible DNA regions by probing accessible chromatin with hyperactive mutant Tn5 transposase that inserts sequencing adapters into open regions of the genome (90). Single cell ATAC-seq (scATAC-seq) measures chromatin accessibility enabling marker-free identification of cell type-specific *cis*- and *trans*-regulatory elements and mapping of disease-associated enhancer activity and reconstruction of trajectories of cellular differentiation, and has been used to map gene regulation in cell-to-cell variability and rare cell phenotypes, including in healthy and malignant immune cells (61, 63).

### The Analysis of Single-Cell Data

Single-cell technologies generate huge amount of information that allow the exploration of cellular diversity at unprecedented depth and throughput. For this reason, one of the major analytical challenge is how to visualize and understand this high-dimensional datasets originating from high dimensional flow cytometry, scRNA-seq and scATAC-seq. Data generated by high-dimensional flow cytometry (up to 30 parameters in several million cells) can no longer be analyzed by using classical manual analysis techniques involving the use of bidimensional dot plots (91). Manual gating analyses is hard to reproduce, as is subjective and biased, and for large data set is extremely time consuming. Large datasets are computationally demanding, and therefore require the development and the application of novel techniques.

Computational flow cytometry provides a set of packages to analyze and visualize large amount of cells in an unbiased manner (92). These tools are automated, meaning that the quality of data is fundamental to get rid of false positive. For this reason, before analyzing high-parameter flow cytometry datasets, files need to be perfectly compensated, cleaned from the presence of aggregates and turbulences during acquisition. Only after this step, data can be analyzed by unsupervised tools (93, 94).

**Figure 1:**
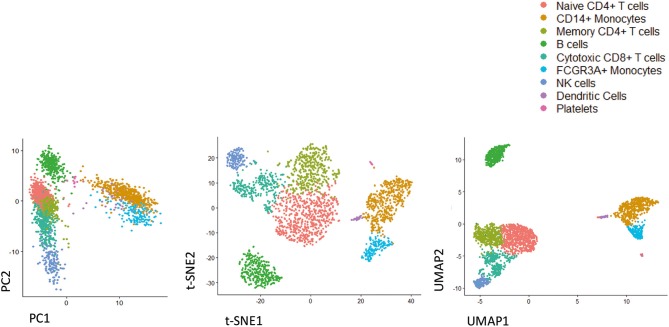
Representative image of different dimensionality reduction techniques that are widely used in single cell studies. As shown by analyzing freely available scRNA-seq dataset (3k PBMC, from 10X Genomics), UMAP preserves much of the local and more of the global data structure, highlighting its ability to resolve even subtly differing cell population. From left to right PCA, t-SNE, UMAP.

scRNA-seq requires pre-processing of data based on quality control performance and alignment (78). Several efforts have been made from bioinformaticians to develop and optimize new software and packages able to provide insights on the complex biology and dynamics of cells (66). Most software provide information regarding identification and characterization of cell types and their spatial organization in time (78). A canonical pipeline of data analysis firstly requires data visualization. There are methods based on dimensionality reduction techniques, including principal component analysis (PCA), t-distributed stochastic neighbor embedding (t-SNE), One-Dimensional Soli-Expression by Nonlinear Stochastic Embedding (ONE-sense), Uniform Manifold Approximation and Projection (UMAP), that aim to preserve the main structure of data while reducing a high-dimensional data description to a lower-dimensional projection (95–97). An example of the analysis of the same data by using PCA, t-SNE, and UMAP is reported in Figure 1. In addition, clustering-based techniques are available that group cells into cell type clusters in the original, high-dimensional space and subsequently use visualization algorithms to represent these cell type clusters in a lower-dimensional space (93, 98, 99).

Secondly, differences in gene expression level between populations need to be analyzed. To this purpose, specialized methods have been designed for single-cell data that considers single cell features such as technical dropouts and shape of the distribution (100).

Thirdly, the software Monocle and Wanderlust independently introduced the concept of “pseudotemporal analysis,” in which scRNA-seq data are collected from a population of cells undergoing a dynamic biological process and then computationally ordered into a trajectory that reflects the continuous changes in gene expression that occur from the beginning to the end of the process (101–103). Pseudotime trajectories allow to identify genes that exhibit differential expression over the course of the biological process and cluster them based on their expression dynamics. As of February 2020, more than seventy trajectory inference tools have already been developed (104).

## The Workflow of Single-Cell Experiment

**Figure 2:**
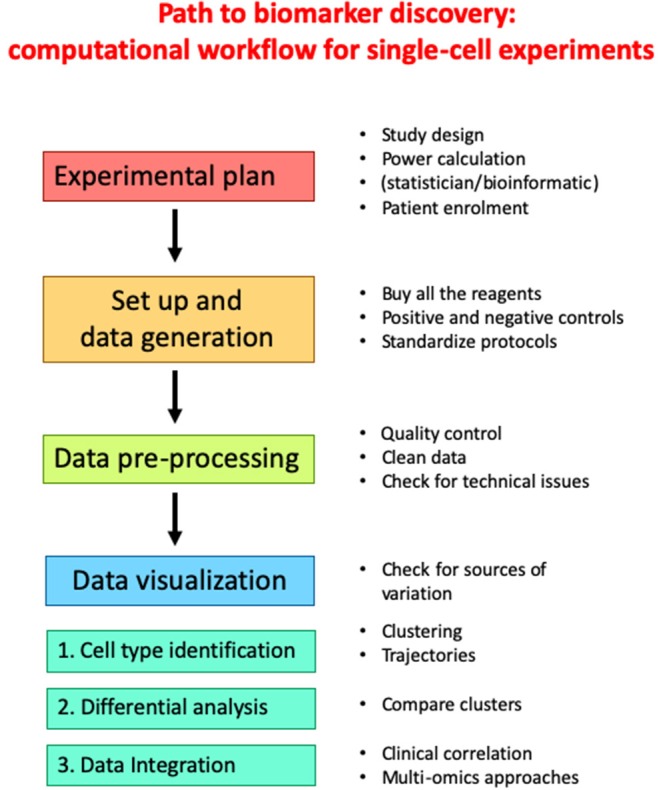
Workflow for canonical single-cell experiment.

Regardless of the specific technology employed to generate a particular dataset, a common workflow can be formulated which involves multiple steps linking the initial study design to the final correlation to clinical data. A typical pipeline for single-cell experiments is reported in Figure 2. An accurate experimental planning is imperative to avoid technical issues and improve scientific reproducibility. Several professionals, including the statistician, the bioinformatician, the biologist and the clinician should be involved at this step to: (i) define the biological question; (ii) find patients; (iii) calculate the sample size; (iv) define the number of replicates; (v) decide the number of cells; (vi) define the sequencing depth (in the case of scRNA-seq or sc-ATACseq experiments); (vii) choose the appropriate equipment (105). At this stage, experimental protocols should be standardized, and appropriate positive and negative controls should be selected to ensure good quality results. Then, experiment is performed and raw data are generated. Alongside, data pre-processing is performed. Quality control involves the examination of data, their possible transformation and normalization, the check for technical issues, batch effects or unexpected results. At the end of the entire process, clean data need to be visualized and analyzed by computational approaches to identify clusters and trajectories, and potentially derive novel predictive biomarkers of response to ICI.

## Data From Single-Cell Studies

### Immune Cells in the Tumor Microenvironment

Tumors contain different cell populations in endless evolution. This diversity is commonly referred to as tumor heterogeneity, and is considered the main driver of resistance to therapy and metastasis (106). The full comprehension of this heterogeneity would be extremely important to optimize existing therapeutic intervention and find new strategies to break down relapses and mortality. The recent development of technologies based on sequencing individual cells has been crucial to address tumor heterogeneity and to elucidate how cells are organized into multicellular systems. Single cell profiles not only revealed that human tumors comprise subpopulations of genetically different diverse malignant cells, but also that a profusion of different cell types from the surrounding tissues and the immune system, each with a precise role in pathogenicity, is present within the TME (106, 107). The immune components of the tumor microenvironment in different kind of malignancies, including non-small cell lung cancer (NSCLC), clear cell RCC (ccRCC), breast cancer (BC), HCC, glioblastoma multiforme (GMB), microsatellite instability-stable CRC have been recently annotated and finely characterized (88, 108–111). In general, in the majority of these tumors, immune cells were mostly T cells, whereas myeloid cells, B cells and NK cells were found at lower frequencies (108–111). Only GMB had higher levels of CD68+ myeloid cells if compared to T cells (88).

In NSCLC tumor samples, different subsets of CD8+ T cells, conventional CD4+ T cells, and Treg cells have been found (109). Each subset is characterized by a precise gene expression signature, which reflects a specific functional status. Two main clusters were found at high frequency: (i) exhausted CD8+ T cells, characterized by increased expression of effector molecules, including GZM-A, granulysin (GNLY), perforin (PRF), GZM-B, NKG7, and inhibitory receptors, like lymphocyte activating (LAG)-3 (CD223), T cell immunoreceptor with Ig and ITIM domains (TIGIT), PD-1, and CTLA-4; (ii) suppressive CD4+ Treg cells, characterized by increased expression of costimulatory molecules, including CD28 and inducible T cell costimulatory (ICOS or CD278), and inhibitory receptors like CTLA-4 and TIGIT (109). Moreover, two CD8+ T cell subsets exhibited a functional state that precede exhaustion, and is indeed called pre-exhaustion state. These subsets do not express CTLA-4, and express mild levels of TIGIT, PD-1, and the transcription factor TOX, which is a critical driver of exhaustion (112–115). Whether or not pre-exhausted subsets could be more effectively reinvigorated by ICI than fully exhausted subsets is still not known. Furthermore, the expression level of PD-1 or other inhibitory receptors does not necessarily correlate with exhaustion-dependent dysfunction. It was indeed reported that tissue-resident memory T (T_RM_) cells expressing PD-1, T-Cell Immunoglobulin And Mucin Domain-Containing Protein-3 (TIM-3) and negative for CD127 (the α chain of the IL-7 receptor), which are present in lung tumors, proliferate, can upregulate TCR activation–induced genes, exhibit a transcriptional signature indicative of effector, survival, and tissue-residency properties, and produce cytokines, like IL-2, IFN-γ and TNF-α (116). In early-stage triple-negative BC, among CD8+ T cells infiltrating the TME, T_RM_ cells display high levels of genes encoding for cytotoxic molecules, including *GZMB* and *PRF1*, high levels of genes encoding for inhibitory checkpoint, as well as high levels of genes associated with proliferation (117). This means that the expression of inhibitory receptors is not a unique feature of exhausted T cells as several highly functional effector cells also express those receptors. This also means that exhausted T cells are heterogenous, and that T cell exhaustion, as well as T cell dysfunctionality, is a gradual, rather than a discrete, state (118).

In ccRCC, in-depth immunophenotyping analysis identified the main immune cell types in both T cells and tumor-associated macrophages (TAMs) (108). Concerning T cells, eight CD4+ clusters, eleven CD8+ clusters, one CD4+/CD8+ double positive cluster, and one CD4–/CD8– double negative cluster were identified (108). Notably, whereas PD-1 had a broad expression both CD8+ and CD4+ T cell population, other inhibitory molecules, like TIM-3, CTLA-4, and 4-1BB (CD137) were expressed only by a few PD-1+ subsets, indicating that a pre-exhaustion status is also present in ccRCC (108). Interestingly, both in CD8+ and CD4+ T cells, PD-1 is co-expressed with CD38, which mediates immunosuppression by activating nitric oxide synthetase which in turn catalyzes the production of nitric oxide from arginine. Although CD38 has traditionally been linked to T cell activation, these data suggest that its expression is not restricted to activated cells, but rather can be extended to exhausted T cells, at least in ccRCC and BC (108, 110). In the latter, a higher frequency of PD-1^high^CTLA-4+CD38+ T cells has been observed in tumor biopsies if compared to juxta-tumoral tissues, thus confirming that PD-1 and CD38 are both expressed in exhausted cells (110). Indeed, CD8+ T cells expressing high level of PD-1 also expressed the co-inhibitory molecules TIM-3 and CTLA-4, and the activation markers HLA-DR and CD38, which were not expressed by CD8+ T cells expressing intermediate levels of PD-1 (110). Similarly, HCC biopsies were mostly enriched by exhausted CD8+ T cells and Treg cells, and exhausted CD8+ T cells were increased in patients with late stage HCC if compared with early stage HCC (111).

Another cluster of special interest in HCC consisted of mucosal-associated invariant T (MAIT) cells, which are mainly involved in the protection against bacterial or viral mucosal infections (119). Although MAIT cells are considered as a first line defense in the liver, their role in liver cancer is still totally unexplored. Recent evidences revealed that tumor initiation and metastasis formation is reduced in mice knockout for MHC class I-related protein-1 (MR1), which is essential for MAIT development (120). A fraction of MAIT cells among tumor CD8+ T cells has been found also in NSCLC and CRC (109, 121). Interestingly, at least in chronic infections, MAIT cells can express inhibitory receptors, including PD-1, thus meaning that they could also be targets of ICI (122).

In uveal melanoma, single-cell analysis revealed that tumor-infiltrating immune cells, including CD8+ T cells and NK cells, mainly express LAG3, rather than PD-1 or CTLA-4 (123), thus partially explaining the limited efficacy of checkpoint inhibitor therapy in this type of tumors (124). This further confirms that PD-1 is not the exclusive determinant of CD8+ T exhaustion and that the expression of additional markers should be considered across different tumors. The situation is even further complicated by the fact that T cell exhaustion is associated with vast changes in chromatin accessibility (125). Emerging evidence revealed that exhausted CD8+ T cells are epigenetically distinct from functional memory CD8+ T cells, thus suggesting that exhausted T cells occupy a different differentiation state if compared to memory T cells (125).

ScRNA-seq analysis of NK cells obtained from human melanoma metastases indicated that seven clusters of tumor-infiltrating NK are present in these tissues, each with an individual functional specialization (126). NK cells were recently shown to recruit cross-presenting DCs to tumors that are critical for CD8+ T cell–mediated tumor immunity (126).

Although T cells have a dominant role in controlling cancer growth, there is growing interest for other subsets of immune elements that infiltrate the TME, including B and myeloid cells, and that could have a role in the response to therapy. Tumor-infiltrating myeloid cells (TIMs) consist of various subsets of granulocytes, monocytes, macrophages and DCs, at different stage of differentiation, that contribute to cancer progression and response to therapy (127, 128). Among TIMs, the frequency of a specific subset of monocytes, i.e., CD14+, CD16–, HLA-DR^high^ monocytes, has been identified as predictor of progression-free and overall survival in patients with metastatic melanoma prior anti-PD-1 therapy (129). High-dimensional single-cell profiling of lung cancer revealed that an enrichment of macrophages expressing high levels of peroxisome proliferator-activated receptor (PPAR)-γ has been observed in lung adenocarcinoma at early stage (130). Macrophages in the TME have also been studied in breast cancer, renal cancer and HCC using scRNA-seq data (4, 108, 131). TAM-like macrophages in HCC highly express two genes, *SLC40A1* and *GPNMB*. The former encodes ferroportin, a transporter exporting iron from cells, and regulates pro-inflammatory cytokines, like IL-6, IL-23, and IL-1β, through a Toll like receptor (TLR)-dependent pathway (131).

Single-cell profiling of tumor biopsies also revealed that DCs can be present at the TME (4, 108, 130). Among TIMs, DCs are the best armed to prime and activate T cells (132), and among DCs, several subsets with a specific molecular signature have been found to be depleted or enriched in the TME. This was possible by combining CyTOF with single-cell transcriptomics. For example, CD141+ DCs express high levels of *CD207, CLEC9A*, and *XCR1* and preferentially interact with CD8+ T cells, whereas CD1c+ DCs express high levels of *CX3CR1, IRF4, CCL22*, and *CCL17*, which are involved in chemokine signaling, and are better equipped to interact with CD4+ T cells (130). Also LAMP3+ DCs have various interesting features (131). They indeed exhibit a higher migration capacity toward lymph node if compared to conventional DCs (131).

### Checkpoint Inhibitor Therapy Effects on TILs and PBMCs

During the last years, single cell technologies have been used to interrogate a number of tumoral settings with the goal to understand both successful and ineffective immune responses after treatment with ICI, and identify accessible biomarkers that clinicians could use to discriminate between patients who most likely respond or not to therapy (2). The most important studies reporting the use of cutting-edge single-cell technologies to identify the effects of checkpoint inhibitor therapy on immune system are reported in Table 2. Concerning the type of neoplasia, the vast majority of studies regard patients with melanoma or NSCLC, treated with anti-CTLA-4 or anti-PD-1 or, in few cases, with both of them.

**Table 2:** Main studies reporting the use of cutting-edge single-cell technologies to identify the effects of checkpoint inhibitor therapy on immune system.

**Tumor type**	**Sample source**	**Technology**	**Main findings**	**References**
Melanoma	TILs	Flow cytometry	• High level of CD8+, PD-1^++^, CTLA-4^++^ TILs correlated with response to therapy and progression-free survival; • Functional analysis of these cells revealed a partially exhausted T cell phenotype; • Assessment of metastatic lesions during anti–PD-1 therapy demonstrated a release of T cell exhaustion, as measured by an accumulation of highly activated CD8+ T cells within tumors.	(133)
Melanoma	PBMCs TILs	Flow cytometry	• CD8+ T cells responding to therapy display an exhausted phenotype; • TIL clones in responding peripheral blood CD8+ T cell population and blood Ki67+, CD8+ T cell response correlates with tumor burden.	(134)
NSCLC	PBMCs	Flow cytometry	• Increase in Ki67+, PD-1+, CD8+ T cells following therapy in ~70% of patients (after the first or second treatment cycle); • Effector-like phenotype (HLA-DR+, CD38+, Bcl-2^low^), expressed costimulatory molecules (CD28, CD27, ICOS), and had high levels of PD-1 and coexpression of CTLA-4.	(135)
Melanoma	TILs	Mass cytometry; RNA-seq	• The CD8+ T cell population expanded in ICI-treated tumors displayed a CD45R0+, PD-1+, TBET+, EOMES+ phenotype; • CTLA-4 blockade induces expansion of ICOS+ Th1-like CD4+ T cells.	(136)
Melanoma	tumor	RNA-seq; scRNA-seq; *in situ* multiplex protein	• Resistance program expressed by malignant cells, associated with T cell exclusion and immune evasion. The program is expressed prior to immunotherapy, characterizes cold niches *in situ*, and predicts clinical responses therapy; • CDK4/6-inhibition represses this program in individual malignant cells, induces senescence, and reduces melanoma tumor outgrowth in mouse models *in vivo* when given in combination with immunotherapy.	(137)
NSCLC	TILs	Flow cytometry RNA-seq	• PD-1^++^ T cells showed a markedly different transcriptional and metabolic profile from PD-1^+−^ and PD-1^−^ lymphocytes, as well as an intrinsically high capacity for tumor recognition; • PD-1^++^ lymphocytes were impaired in classical effector cytokine production, they produced CXCL13, which mediates immune cell recruitment to tertiary lymphoid structures; • The presence of PD-1^++^ cells was strongly predictive for both response and survival.	(138)
Melanoma	tumor	scRNA-seq; ATAC-seq	• Two distinct states of CD8+ T cells were defined by clustering and associated with patient tumor regression or progression; • TCF7 was visualized within CD8+ T cells in fixed tumor samples and predicted positive clinical outcome.	(139)
Melanoma 1 patient, 90 years old	PBMCs TILs	Flow cytometry; TCR sequencing	• Proliferating CD8+ T cells exhibited an effector-like phenotype with expression of CD38, HLA-DR and Granzyme B, as well as expression of the positive costimulatory molecules CD28 and CD27; • TCR sequencing of peripheral blood CD8+ T cells revealed a highly oligoclonal repertoire at baseline with one clonotype accounting for 30%.	(140)
Melanoma	PBMCs	Mass cytometry	• Frequency of CD14+, CD16–, HLA-DR^hi^ monocytes before therapy is a strong predictor of progression-free and overall survival.	(129)
Melanoma, NSCLC	TILs PBMCs	Flow cytometry; RNA-seq	• CD4+, FoxP3-, PD-1^hi^ T cells (4PD1^hi^, a TFH-like phenotype) negatively regulate T cell responses; • CTLA-4 and PD-1 blockade modulate 4PD1^hi^ frequency in opposing directions; • 4PD1^hi^ are a pharmacodynamic and negative prognostic factor of checkpoint blockade.	(141)
Melanoma, Prostate cancer, Bladder cancer	Tumor	IHC; CyTOF	• Both ipilimumab and tremelimumab increase the infiltration of CD4+ and CD8+ cells without significantly changing or depleting FOXP3 cells within the tumor microenvironment.	(42)
Melanoma	Tumor	RNA-seq; Multiplex IHC; CyTOF	• Tumors from non-responders to monotherapy often express other immune checkpoints and higher gene expression profile of EOMES+, CD69+, CD45RO+ T cells is associated with greater tumor shrinkage in both therapies.	(142)
Glioblastoma	Tumor TILs	Flow cytometry; RNA-seq	• Neoadjuvant nivolumab resulted in enhanced expression of chemokine transcripts, higher immune cell infiltration and augmented TCR clonal diversity among tumor-infiltrating T lymphocytes.	(143)
Melanoma	Tumor	MARS-seq; scTCR-seq	• scRNA-seq and TCR analysis in melanoma identifies a gradient of T cell dysfunction; • Cytotoxic T cells are unconnected to the dysfunctional gradient; • Proliferation in CD8+ T cells is most profound during early stages of dysfunction; • The abundance of dysfunctional CD8+ T cells is associated with tumor recognition.	(144)
Melanoma	TILs PBMCs	Flow cytometry; RNA-seq	• After a single dose of anti-PD-1, rapid pathologic and clinical responses associated with accumulation of exhausted CD8+ T cells in the tumor at 3 weeks, with reinvigoration in the blood observed as early as 1 week; • A pre-treatment immune signature (neoadjuvant response signature) associated with clinical benefit.	(145)
Melanoma	TILs	scRNA-seq; TCR sequencing	• Tracking TCR clones and transcriptional phenotypes revealed coupling of tumor recognition, clonal expansion and T cell dysfunction marked by clonal expansion of CD8+, CD39+ T cells; • The expanded clones consisted of novel clonotypes that had not previously been observed in the same tumor. Clonal replacement of T cells was preferentially observed in exhausted CD8+ T cells and evident in patients with basal or squamous cell carcinoma.	(146)
Basal cell carcinoma	PBMCs TILs	scATAC-seq	• Serial tumor biopsies before and after PD-1 blockade identifies chromatin regulators of therapy-responsive T cell subsets and reveals a shared regulatory program that governs intratumoral CD8+ T cell exhaustion and CD4+ T follicular helper cell development.	(63)
Melanoma, RCC	TILs	scRNA-seq; CyTOF	• B cells found in tumors of responders; • B cells localized in the TLSs; • CyTOF shows differential B cell phenotypes.	(147)

*TILs, Tumor-infiltrating lymphocytes; PBMCs, peripheral blood mononuclear cells; RNA-seq, RNA sequencing; scRNA-seq, single-cell RNA sequencing; NSCLC, non-small cell lung cancer; TCR, T cell receptor; CyTOF, Cytometry by Time-Of-Flight; MARS-seq, massively parallel single-cell RNA-sequencing; scATAC-seq, single-cell Assay for Transposase-Accessible Chromatin using sequencing; ICI, immune checkpoint inhibitors; TLSs, tertiary lymphoid structures; IHC, immunohistochemistry*.

#### CD8+ T Cells

Overall, among immune cells, main differences have been found in T cell compartment, and among T lymphocytes, cytotoxic cells are often affected by checkpoint inhibitor therapy. Single-cell technologies have shown that cytotoxic T cells do not form a homogenous population but are a heterogenous mix of cells with different transcriptomes, phenotype and functional capacity. According to their differentiation state and on the basis of the expression levels of few proteins, CD8+ T cells have been typically classified in well-defined subsets of naïve, memory, and effector cells (148). During the last few years, high-dimensional single-cell profiling allowed immunologists to understand that a variety of other states with significant phenotypic and functional diversity is observed within the CD8+ T cell compartment (149). This heterogeneity becomes increasingly relevant at the level of the TME, both within and among patients, and could be at the basis of the mechanisms linking T cells states and response to checkpoint inhibitor therapy.

A study performed on freshly isolated metastatic melanoma samples from two cohorts of 20 patients used flow cytometry alone to show that an increased fraction of tumor-infiltrating CD8+ T cells expressing high level of PD-1 and CTLA-4 strongly correlated with response to therapy and progression-free survival (133). These cells were named as “partially exhausted,” as they retained the capacity to produce IFN-y but lose the ability to produce TNF-α and IL-2 (133). In another cohort of patients with melanoma treated with ICI, single-cell RNA profiling of immune cells from baseline, on-therapy and post-therapy tumor samples was performed (139). Exhausted cells were defined as those with increased expression of several genes encoding for inhibitory receptors, including *LAG3, FASLG, HAVCR2* (which encodes for TIM-3), *PDCD1* (which encodes for PD-1), *CD38* (139). It was also showed that TIM-3 and CD39 were markers for discriminating exhausted from memory CD8+ T cells, and that the elevated frequency of TCF7+, CD8+ T cells can predict with a positive outcome (139). Concerning CD39, it was also found that CD8+ TILs from lung cancer and CRC were not only specific for tumor antigens but also could recognize a broad range of epitopes unrelated to cancer, and that CD39 was critical to distinguish tumor-specific CD8+ TILs from bystander CD8+ T cells (150).

In other melanoma patients treated with anti-PD-1, the combination of scRNA-seq to TCR-seq allowed to identify a dysfunctional axis consisting of cells able to actively proliferate despite having an “exhausted” phenotype (144). The application of different single-cell technologies to three different cohorts of melanoma patients treated with anti-PD-1 allowed to understand that a noteworthy phenotypic heterogeneity is observed within CD8+ TILs that display characteristics of dysfunction, reflected by various combinations and expression levels of inhibitory receptor and activation markers, the proliferative capacity and the ability to produce cytokines and effector molecules. A resistance program that is associated with hallmarks of T cell exclusion and suppression has also been found in malignant cells prior to immunotherapy, likely indicating the presence of intrinsic resistance (137).

Other striking results of single-cell technologies have been obtained in blood samples from cancer patients treated with ICI. In those with melanoma, circulating Ki67+, CD8+ T cell response was correlated with tumor burden (134). Similar results were found in NSCLC treated with anti-PD-1. After therapy, an increase of Ki-67+, PD-1+, CD8+ T cells displaying an effector-like phenotype (HLA-DR+, CD38+, Bcl-2^low^), costimulatory molecules (CD28+, CD27+, ICOS+), high levels of PD-1 and co-expression of CTLA-4 was observed in patients responding to therapy (135, 140). In the same patients, the expansion of CD39+, CD8+ T cells was observed a few days after a single dose of anti-PD-1 in a neoadjuvant setting (145). Tracking TCR clones and transcriptional phenotypes in basal cell carcinoma (BCC) also revealed clonal expansion of CD8+, CD39+ T cells, which co-expressed markers of chronic T cell activation and exhaustion. However, in this case, the expansion of T cell clones did not derive from pre-existing TILs, but from novel clonotypes that had not previously been observed in the same tumor (146). This suggests that the response to anti-PD-1 depends on the intrinsic capacity of tumors to recruit novel T cell clones, which replace pre-existing exhausted T cells that have insufficient capacity to reinvigorate in response to therapy (151).

In addition, data obtained from melanoma samples and peripheral blood from patients treated with anti-CTLA-4 and anti-PD-1 revealed that treatment-specific effects can be observed. Indeed, while anti-PD-1 mainly induced the expansion of specific tumor-infiltrating exhausted-like CD8+ T cell subsets, anti-CTLA-4 led to the expansion of an ICOS+ Th1-like CD4+ effector subsets other than engaging specific subsets of exhausted-like CD8 T cells (136). It was also reported that the population of CD8+, CD45RO+, PD-1+, TBET+, EOMES+ T cells increased after treatment only in TILs if compared to the peripheral blood (136), and that the gene expression signature of EOMES+, CD69+, CD45RO+ T cells was associated with greater tumor shrinkage in both therapies (142). Likewise, in a cohort of patients with NSCLC treated with anti-PD-1, the presence of PD-1^++^ T cells within the tumor was strongly predictive for both response and survival (138). PD-1^++^ T cells indeed produce C-X-C Motif Chemokine Ligand 13 (CXCL13), which mediates immune cell recruitment to TLSs (138). Similarly, in a cohort of patients with GMB treated with anti-PD-1 an enhanced expression of chemokine transcripts, higher immune cell infiltration and augmented TCR clonal diversity among tumor-infiltrating TILs was reported (143). In summary, a large variability can be observed among different patients' cohorts concerning the abundance of different T cell functional states. An increase in CD8+ T cells with an effector-like phenotype expressing inhibitory/costimulatory molecules and proliferations markers has been described in several cancer settings after therapy with ICI. However, only in few cases this immune cellular response were correlated with a measurable clinical response.

#### CD4+ T Cells

The vast majority of recent studies based on single-cell technologies have been focused on CD8+ T cells, as their role in cancer surveillance, editing and control is compelling. However, a role in tumor control is also played by the CD4+ T cell compartment, as reflected by the observation that CD4+ T cells infiltrate the tumor, and by the prognostic value of several CD4 subsets in different malignancies (152–154). Distinct CD4+ T cells subsets have been described by means of single-cell technologies, including naïve cells, memory-like cells, Th1 cells, Treg, follicular helper T cells (T_FH_), and even cytotoxic effector T cells (109, 111, 121, 141, 144, 146, 155, 156).

In NSCLC tumor and blood samples, scRNA-seq allowed to identify seven CD4+ T cell populations (109). Interestingly, among them an “exhausted” CD4+ T subset was present and displayed a gene signature comparable to that observed in exhausted CD8+ T cells. Two Treg clusters were also identified and one of them was defined as “suppressive Treg” as cells expressed high levels of *TNFRSF9* (encoding for 4-IBB), *TIGIT* and *CTLA-4* genes (109). A closer quantification of this cluster in blood and tumor samples revealed that a higher percentage of suppressive Treg cells was present in tumor if compared to blood (109).

A combination of scRNA-seq and TCR analysis allowed to identify a subset of “dysfunctional” CD4+ T cells in a cohort of melanoma patients, and again these dysfunctional cells expressed specific combinations of genes encoding for inhibitory checkpoints that partially overlapped with those observed in CD8+ T cells (144). The fact that in TME CD4+ T cell also express PD-1 and/or CTLA-4 suggests that most of the current immunotherapy strategies that use checkpoint inhibitor can potentially leverage on these cells. Although data dissecting the effects of these drugs on CD4+ T cells are still elusive, it was recently found that in melanoma patients treated with anti-PD-1/anti-CTLA4 the frequency of the T cell population characterized by a T_FH_-like phenotype (CD4+, Foxp3-, PD-1^high^) is modulated differently by the two drugs and is a negative prognostic factor of response to therapy (141).

#### Other Cells Than T Lymphocytes

Through mass cytometry and scRNA-seq, in GMB a unique subset of macrophages expressing high levels of CD73 able to persist after anti-PD-1 therapy was observed (88). Notably, a number of reports have shown that CD73 can induce immunosuppression in GMB (157, 158).

Tumor-infiltrating B cells exist and are mainly found in lymphoid aggregates, known as TLSs (147, 159). It was found that the density of CD20+ B cells and TLSs, together with the ratio of TLSs to tumor area were higher in responders than in non-responders (147). Moreover, a prognostic B-cell-related gene signature was found in patients with cutaneous melanoma or RCC. Several genes, including *FCRL5, IDO1, IFNG*, and *BTLA*, were indeed enriched in patients responding to therapy (147).

## Conclusions

The interactions between tumor and immune system are ruled by several complex mechanisms, with several main players such as malignant cells, tumor infiltrate, tumor stroma and vasculature, and systemic factors. Among them, the heterogeneity of intra-tumor immune cells has been extensively studied by using traditional approaches, including basic flow cytometry and immunohistochemistry, which have the limitations described above. Recently, substantial advances in emerging techniques and bioinformatic pipelines have enabled researchers to investigate in detail the complexity of the TME, and to interrogate in depth previously unexplored cell types. Among single-cell approaches, scRNA-seq has been crucial for exploratory analysis, and the combination of scRNA-seq with mass cytometry has been even more helpful.

The application of single-cell technologies to tumor and blood samples has generated and will generate in the upcoming years, an explosion of new data with a clear impact in the translational clinical research, thanks to the identification of possible biomarkers. It is likely that the huge amount of information will also thoroughly revolutionize the field of basic research in immunology and cancer biology. A big effort should be posed to make all data, including the raw ones, available to the scientific community and to create rigs for data extraction. The information gathered from these technologies will add novel hallmarks of response to immune therapy that could be integrated in the routine clinical management.

Nonetheless, the route to the discovery of new biomarkers is still bumpy. Due to the high sensitivity of single-cell technologies, adequate attention must be put into experimental setup and execution. A very careful handling of cells during pre-processing and an adequate data analysis with potent bioinformatic tools are critical factors to preserve the native biological profile that will ensure meaningful conclusions.

Lastly but importantly, although a number of specific immune cell subsets have been identified that are associated with response or resistance to ICI, still additional studies should be planned to address the role and function of different types of immune cells in the TME. Investigating the role of T cell exhaustion and/or dysfunction in the TME and translating this knowledge to clinical practice can be considered main challenges in the battle against cancer.

## Author Contributions

CP, RD, GP, RS, and AC outlined the concept, wrote the manuscript, and overviewed the review. LG and SD wrote the manuscript. LG, SD, and DL designed and prepared the figures.

### Conflict of Interest

The authors declare that the research was conducted in the absence of any commercial or financial relationships that could be construed as a potential conflict of interest.
